# Clinical Efficacy and Safety of Shashen Maidong Decoction in the Treatment of Pediatric Mycoplasma Pneumonia: A Systematic Review and Meta-Analysis

**DOI:** 10.3389/fphar.2021.765656

**Published:** 2021-10-12

**Authors:** Jiawei Wang, Xiao Ma, Shizhang Wei, Tao Yang, Yuling Tong, Manyi Jing, Jianxia Wen, Yanling Zhao

**Affiliations:** ^1^ Department of Pharmacy, The Fifth Medical Center of PLA General Hospital, Beijing, China; ^2^ College of Pharmacy, Chengdu University of Traditional Chinese Medicine, Chengdu, China

**Keywords:** shashen maidong decoction, pediatric *mycoplasma* pneumonia, systematic review, meta-analysis, clinical research

## Abstract

**Objective:** This study was intended to provide data to support the effect of Shashen Maidong Decoction in improving *mycoplasma* pneumonia in pediatric patients through systematic evaluation.

**Methods:** PubMed, the Web of Science, EMbase, CNKI, CQVIP, Wan-Fang, and CBM databases were comprehensively searched from established in June 2021. Randomized controlled trials of TRQI were selected by screening the literature and extracting information. The Cochrane RCT Evaluation Manual was used to evaluate the methodological quality of all included studies, and Meta-analysis was performed using Stata 14.0 and Review Manager 5.4 software.

**Results:** A total of 1,127 patients from 12 clinical studies met the inclusion criteria. Meta-analysis results showed that the treatment group of Shashen Maidong Decoction was able to significantly increase the overall efficiency level and significantly reduce the incidence of adverse reactions, time for disappearance of cough, time for relief of cough, time for defervescence, time for disappearance of lung rales, time for return to normal of chest X-ray, T lymphocyte subpopulation (CD3^+^) and tumor necrosis factor-α (TNF-α) and other index levels (*p* < 0.05).

**Conclusion:** Shashen Maidong Decoction has a significant improvement in the levels of relevant indexes in pediatric *mycoplasma* pneumonia, which provides a basis for the safety and efficacy of pediatric *mycoplasma* pneumonia. However, due to the small sample size included in the study, the study quality was not high, and more randomized controlled trials of high quality are required for further validation.

## Introduction

*Mycoplasma pneumoniae* (MP) is one of the most common causes of community-acquired pneumonia (CAP) in children, and studies have shown that the incidence of pediatric *mycoplasma* pneumonia (MPP) accounts for approximately 25% of all CAP (Lee et al., 2018; Jain et al., 2015; Wang, 2017). MP is a highly evolved and polymorphic bacterial pathogen without a cell wall (Guo et al., 2019). It has a wide range of clinical manifestations, including upper respiratory tract infections, pneumonia, and extrapulmonary manifestations (such as encephalitis) (Kutty et al., 2019). The most common pathological manifestation of MPP is characterized by mononuclear cell infiltration in the bronchi and perivascular areas and thickening of the bronchovascular bundles (Tanaka, 2016). Currently, macrolide antibiotics (MA) such as azithromycin (AZM) are preferred in Western medicine for the treatment of pediatric MMP (Ni, 2019), however, epidemiological results in recent years have shown that resistant MP strains of macrolides are increasing year by year, leading to an increase in the number of critically ill and refractory patients or a prolonged course of disease (Wu and Wu, 2016). Therefore, there is an urgent need to find alternative drugs under the theoretical system of TCM. In the theoretical system of TCM, there is no concept of MP, which is considered by TCM to be related to the delicate lungs, lack of strengthening of the external guard, and sensation of wind evils (Jiang et al., 2017). Chinese medicine is gradually showing the unique advantages of motherland medicine in the treatment of MPP, and MPP can achieve better efficacy after the diagnosis and treatment of Chinese medicine, especially in relieving symptoms, reducing cough, and shortening the course of the disease (Tan and Jiang, 2018). The effectiveness of TCM in the treatment of *mycoplasma* pneumonia has been shown (Shi and Yang, 2019), such as Yangyin Qingfei Decoction (YYQFD) (Lin, 2020) and Shaoyao Gancao Decoction (SGD) (Liu et al., 2020) for chronic cough following *mycoplasma* pneumonia in pediatric patients. The main effects of Shashen Maidong Decoction (SMD), which is derived from the “Article of Warming Diseases” written by Jutong Wu, a famous doctor in the Qing Dynasty, is mainly composed of 7 kinds of Chinese medicinal materials (Table 1), are to generate fluid and moisten dryness and to clear the lung and stomach and is clinically applied to symptoms of warming evil injuring the lung and deficiency of lung yin and heat (Zhou, 2017). Xiaoxia Shi (Shi, 2011) and Jing Xu (Xu, 2012) et al. found the effectiveness of SMD with addition and subtraction in the treatment of *mycoplasma* pneumonia-related conditions. Thus suggesting that SMD could be used as a new treatment for MP in pediatric patients.

**TABLE 1:** Ingredients and basic information of SMD.

Chinese botanical drugs	Latin name	Part of botanical drugs	Ingredient percentage (%)
Gan Cao	*Glycyrrhiza uralensis* Fisch. ex DC	Radix & rhizome	7.90
Yu Zhu	*Polygonatum odoratum* (Mill.) Druce	Rhizome	13.16
Sha Shen	*Adenophora stricta* Miq	Fruit	19.73
Sheng Bian Dou	*Vicia lens* subsp. lens	Seed	13.16
Mai Dong	*Ophiopogon japonicus* (Thunb.) Ker Gawl	Radix	19.73
Tian Hua Fen	*Trichosanthes* kirilowii Maxim	Radix	13.16
Sang Ye	*Morus alba* L	Leaf	13.16

**GRAPHICAL ABSTRACT:**
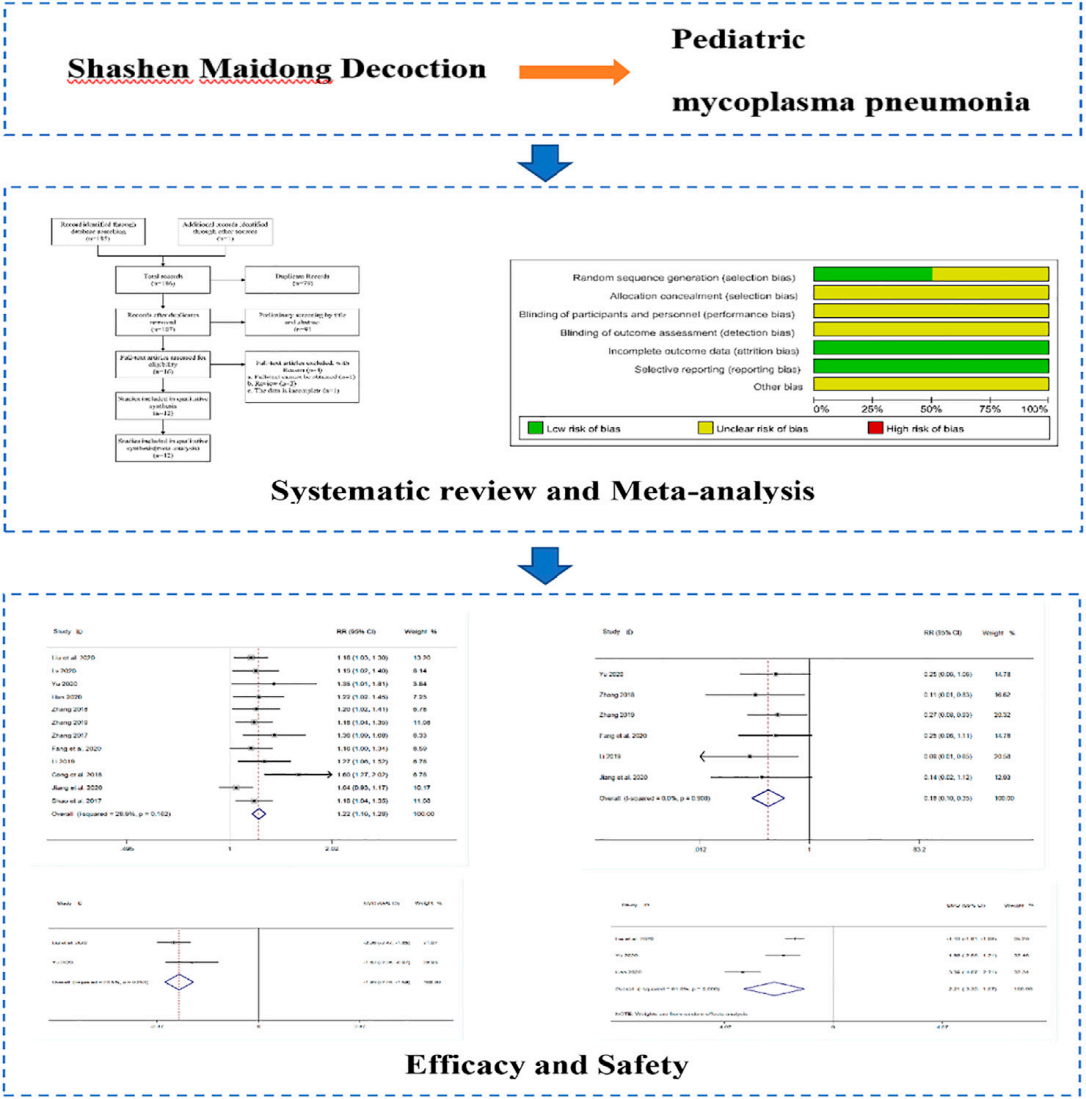


Therefore, this study used the method of Meta-analysis, aiming to systematically analyze the therapeutic effect of SMD on pediatric MPP in clinical practice based on clinical studies and to provide a reference basis for its related subsequent clinical studies.

## Materials and Methods

### Literature Source and Search Strategy

PubMed, Web of Science, EMbase, CNKI, CQVIP, Wan-Fang, and CBM databases were comprehensively searched from established to June 2021. The clinical experimental studies related to SMD, Supplemented of SMD, pediatric *mycoplasma* pneumonia, and *Mycoplasma* pneumonia were searched using a combination of subject terms and free words. The English search terms included: Shashen Maidong Decoction, Shashen Maidong Decoctionrosis, SMD, MMP, *Mycoplasma* pneumonia, MP; the Chinese search terms included: Shashen Maidong Decoction, Supplemented of Shashen Maidong Decoction, pediatric *mycoplasma* pneumonia, *Mycoplasma* pneumonia.

### Inclusion and Exclusion Criteria

#### Study Type

Randomized controlled trial (RCT).

#### Research Subjects

Children with *mycoplasma* pneumonia.

#### Interventions

The experimental group used SMD alone or combined with SMD (such as ACU or SGD); the control group used conventional treatment (such as azithromycin) or conventional treatment + YYQFD.

#### Observation Index

1) total efficiency: If the clinical symptoms and objective indexes of the child disappeared and returned to normal, it was regarded as a cure; if the clinical symptoms and objective indexes of the child improved, it was regarded as an improvement. If the clinical symptoms and objective indexes do not change or are aggravated or there are other side effects, the children can be judged to be in an ineffective state. Total effective rate = (number of cured patients + number of improved patients)/total number of patients× 100%;
2) incidence of adverse reactions;
3) time for relief or disappearance of clinical symptoms: time for disappearance of cough, time for relief of cough, time for disappearance of lung rales, time for defervescence, time for return to normal of chest X-ray;
4) other outcome indicators: T lymphocyte subpopulation (CD3^+^), tumor necrosis factor-α (TNF-α).

#### Exclusion Criteria

The exclusion criteria are as follows: 1) studies with systematic evaluation or Meta-analysis; 2) primary and secondary outcome indicators not included in the full text or insufficient data; 3) incomplete articles and duplicate publications; 4) literature not in Chinese or English; 5) reviews, conference abstracts, and animal experiments.

### Data Extraction

More complete data extraction and collection were performed for all included clinical studies. The following basic data were extracted from all included clinical studies: 1) year of publication and first author’s name; 2) sample size of experimental and control groups; 3) age of children; 4) overall sex ratio; 5) interventions; 6) treatment duration; (g) main outcome indicators. Details of all studies are shown in Table 2.

**TABLE 2:** Basic characteristics of the included studies.

Studies	Cases (T/C)	Age (years)	Sex (M/F)	Intervention (T/C)		Course (day)	Outcome index

Liu et al., 2020	71/70	3–18	NA	CT + SMD	CT + YYQFD	7	①③⑤⑦⑧⑨⑩
Lv (2020)	45/45	3–15	52/38	AZM + SMD	AZM	5–7	①
Yu, (2019)	25/25	2–10	29/21	ACU + SMD	CT	14	①②③④⑤⑦⑧⑨
Han (2020)	41/41	1–12	47/35	AZM + SMD	AZM	15	①③④⑤
Zhang (2018)	37/37	1–13	43/31	AZM + SMD	AZM	21	①②
Zhang (2019)	60/60	2–6	69/51	AZM + SMD	AZM	5	①②
Zhang (2017)	40/40	1–13	48/32	AZM + SMD	AZM	21	①
Fang and Hang, (2020)	46/46	1–11	55/37	AZM + SMD	AZM	14	①②④⑥
Li (2019)	39/39	1–12	38/40	ACU + SMD	CT	21	①②
Cong and Zhu, (2018)	50/50	1–6	55/45	SGD + SMD	CT	NA	①③⑥
Jiang and Hao, (2020)	50/50	NA	55/45	AZM + SMD	AZM	3–5	①②
Shao et al. (2017)	60/60	1–5	63/57	SGD + SMD	MA	12	①

Note: Outcome indexes:①total efficiency; ②incidence of adverse reactions; ③time for disappearance of cough; ④time for defervescence; ⑤time for disappearance of lung rales; ⑥time for relief of cough; ⑦time for return to normal of chest X-ray; ⑧TNF-α; ⑨CD3^+^; ⑩CD4^+^.; Abbreviations: T, experimental group; C, control group; SMD, Shashen Maidong Decoction; M, Male; F, Female; AZM, Azithromycin; SGD, Shaoyao Gancao Decoction; YYQFD, Yangyin Qingfei Decoction; MA, Macrolide Antibiotics; CT, Conventional Therapy; ACU, Acupuncture.

### Quality Assessment

The two researchers who assessed the quality of the literature did so according to the Cochrane Collaboration’s risk of bias criteria (Higgins et al., 2011): random sequence generation (selection bias), allocation concealment (selection bias), investigator and subject blinding (implementation bias), outcome evaluator blinding (measurement bias), incomplete outcome data (follow-up bias), selective outcome reporting (reporting bias), and other biases. Both investigators independently reviewed each study, and the final results were expressed as “yes,” “no,” and “inconclusive,” with “yes " represents low-risk bias, “No” represents high-risk bias, and “Uncertain” represents uncertain risk bias.

### Statistical Analysis

The full Meta-analysis was performed using STATA 14.0 and Revman 5.4. For dichotomous variables, we used 95% confidence intervals (95% CI) to calculate the risk ratio (RR) and 95% CI to calculate the mean difference (MD) for continuous outcomes. The *χ* ^*2*^ test and *I* ^*2*^ test were used to evaluate whether the data were heterogeneous. If *p* < 0.05 or *I* ^*2*^ > 50%, the combined data were considered heterogeneous and a random-effects model was used; otherwise, a fixed-effects model was used. We examined the effect of different sample sizes and dosing regimens on the total effective rate by subgroup analysis. In addition, sensitivity analyses were used to investigate the effect of a high-risk study on the overall Mate analysis. Publication bias was analyzed for all included studies using funnel plots and Egger’s test.

## Result

### Literature Selection Process and Results

**FIGURE 1:**
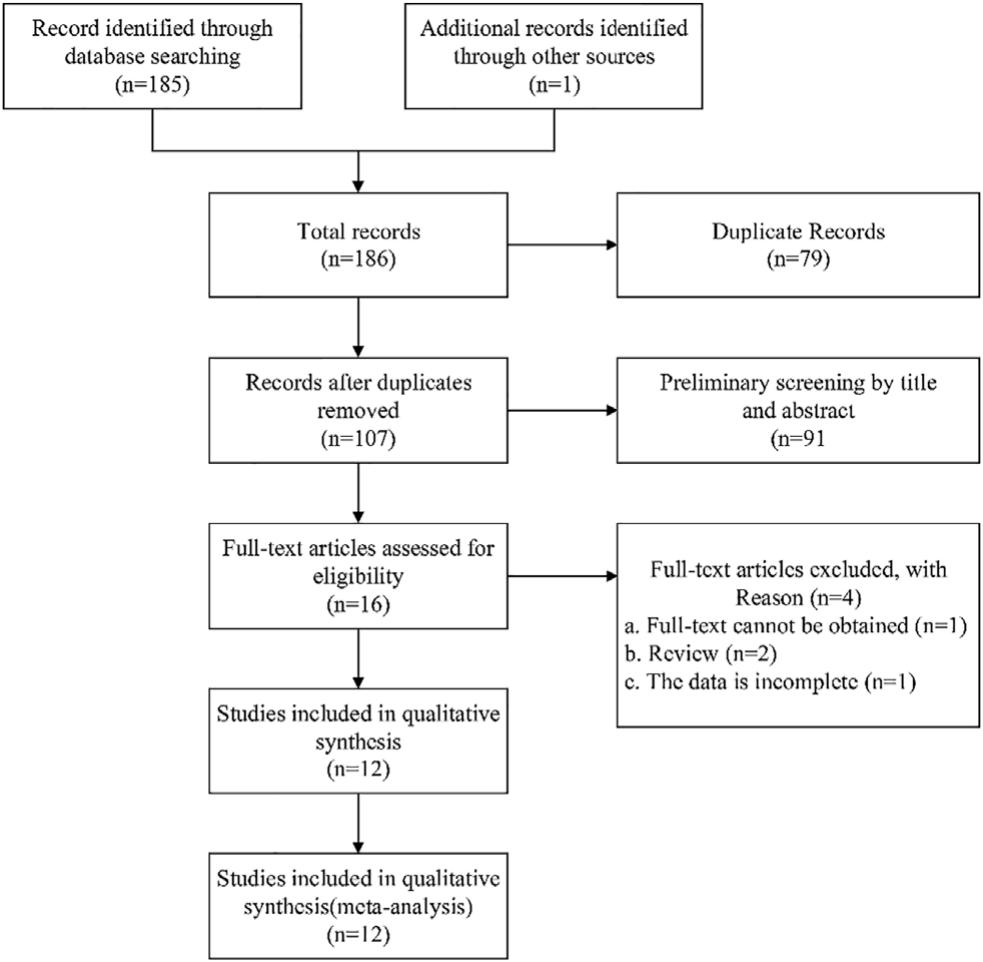
Flow chart of literature screening.

The selection process for selecting eligible studies according to the flow chart is shown in Figure 1. A total of 186 articles were searched through the database, and 107 studies were recorded after removing 79 duplicates. The remaining 16 full-text articles were assessed for eligibility by excluding 91 articles. Among them, one full-text article was not available, two review articles, and one article with incomplete data. Finally, 12 studies that met the inclusion criteria were included (Figure 1).

### Basic Characteristics of the Included Studies

The baseline characteristics of all included studies are shown in Table 2. All studies are randomized controlled trials. A total of 1,127 children participated in 12 studies (Shao et al., 2017; Zhang, 2017; Cong and Zhu, 2018; Zhang, 2018; Li, 2019; Yu, 2019; Zhang, 2019; Fang and Hang, 2020; Han, 2020; Jiang and Hao, 2020; Liu et al., 2020; Lv, 2020), including the experimental group (*n* = 564) and the control group (*n* = 563). The age range of patients is 1–18 years old. The subjects of the study were children suffering from *mycoplasma* pneumonia. A total of 12 studies (Shao et al., 2017; Zhang, 2017; Cong and Zhu, 2018; Zhang, 2018; Li, 2019; Yu, 2019; Zhang, 2019; Fang and Hang, 2020; Han, 2020; Jiang and Hao, 2020; Liu et al., 2020; Lv, 2020) reported the results of clinical efficacy; six studies (Zhang, 2018; Li, 2019; Yu, 2019; Zhang, 2019; Fang and Hang, 2020; Jiang and Hao, 2020) involved adverse reactions; four studies (Cong and Zhu, 2018; Yu, 2019; Han, 2020; Liu et al., 2020) reported the measurement results of the cough disappearance time; three studies (Yu, 2019; Fang and Hang, 2020; Han, 2020) reported the measurement results of the defervescence time; three studies (Yu, 2019; Han, 2020; Liu et al., 2020) reported the measurement results of the disappearance time of rales in the lungs; two studies (Cong and Zhu, 2018; Fang and Hang, 2020) reported the measurement results of the cough relief time; two studies (Yu, 2019; Liu et al., 2020) reported the measurement results of the return to normal of chest X ray time; two studies (Yu, 2019; Liu et al., 2020) Reported the results of the determination of TNF-α; two studies (Yu, 2019; Liu et al., 2020) reported the results of the determination of CD3^+^.

### Risk of Bias of Included Trials

**FIGURE 2:**
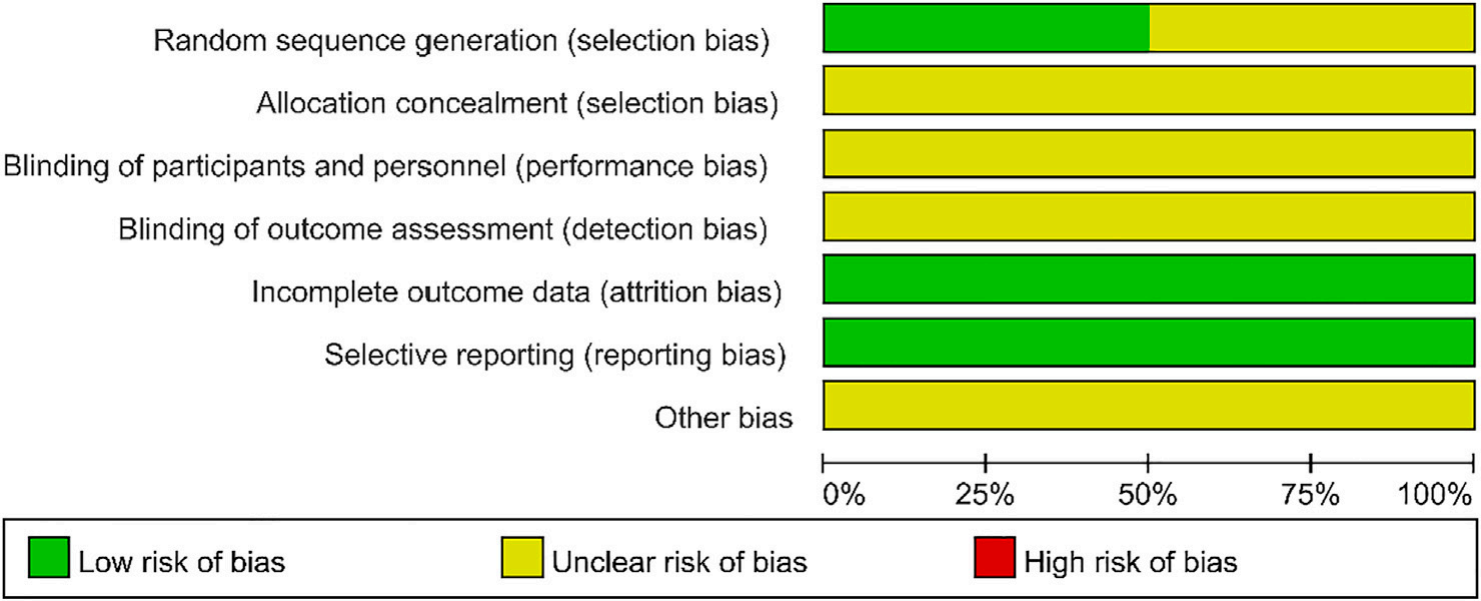
Risk of bias graph.

All 12 studies were randomized controlled trials. Six studies (Liu et al., 2020; Zhang, 2018; Zhang, 2019; Zhang, 2017; Fang and Hang, 2020 et al., 2020; Li, 2019) reported the generation of random sequences: four studies (Zhang, 2017; Li, 2019; Fang and Hang, 2020; Liu et al., 2020) used the random number table method; one study (Zhang, 2018) used the parity number method; one study (Zhang, 2019) used the lottery method. All studies had no incomplete outcome data and no selective outcomes were reported. However, the following four aspects were unclear: allocation concealment; whether investigators and subjects were blinded; whether blinding was imposed by outcome evaluators; and whether other biases were present. The results of the risk of bias assessment for the included experiments are shown in Figure 2.

## Meta-Analysis of SMD in the Treatment of Pediatric Mycoplasma Pneumonia

### Ending Measurement Indicators

#### Effectiveness: Total Efficiency

**FIGURE 3:**
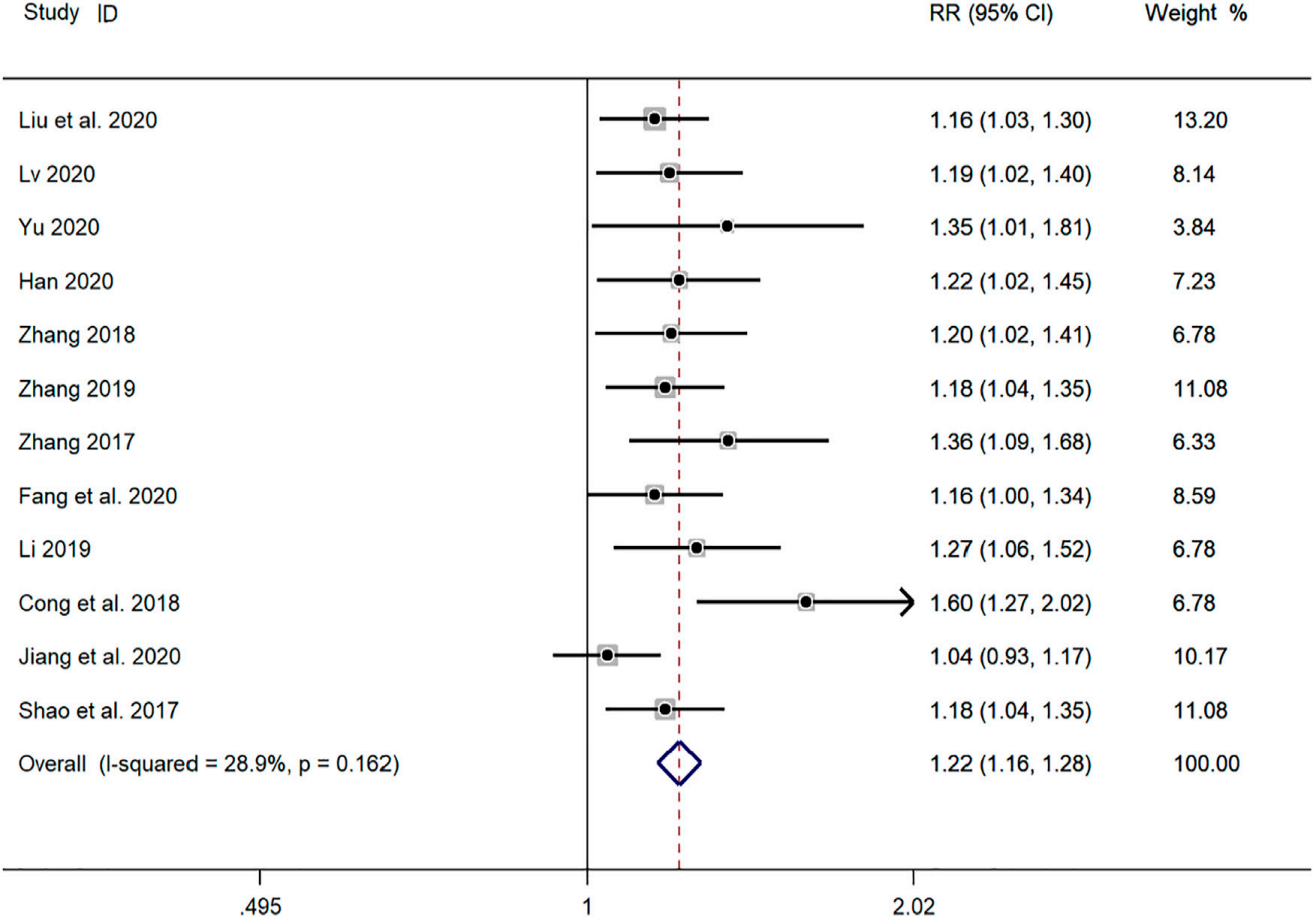
Meta-analysis forest plot comparing the total efficiency level of the experimental group and the control group.

Twelve clinical studies (Shao et al., 2017; Zhang, 2017; Cong and Zhu, 2018; Zhang, 2018; Li, 2019; Yu, 2019; Zhang, 2019; Fang and Hang, 2020; Han, 2020; Jiang and Hao, 2020; Liu et al., 2020; Lv, 2020) reported the total efficiency of SMD in the treatment of *mycoplasma* pneumonia in children. Meta-analysis showed that there was no obvious heterogeneity in the index level of total effective rate (*p* = 0.162, *I* ^*2*^ = 28.9%). Therefore, the fixed effects model is selected for Meta-analysis. The results showed that the total effective rate of the experimental group in the treatment of *mycoplasma* pneumonia in children was compared with that of the control group (*RR* = 1.22, 95%*CI* 1.16–1.28, *p* ≤ 0.001), the difference was statistically significant (Figure 3).

#### Indicators of Relief or Disappearance of Different Symptoms of the Disease

##### Time for Relief of Cough

**FIGURE 4:**
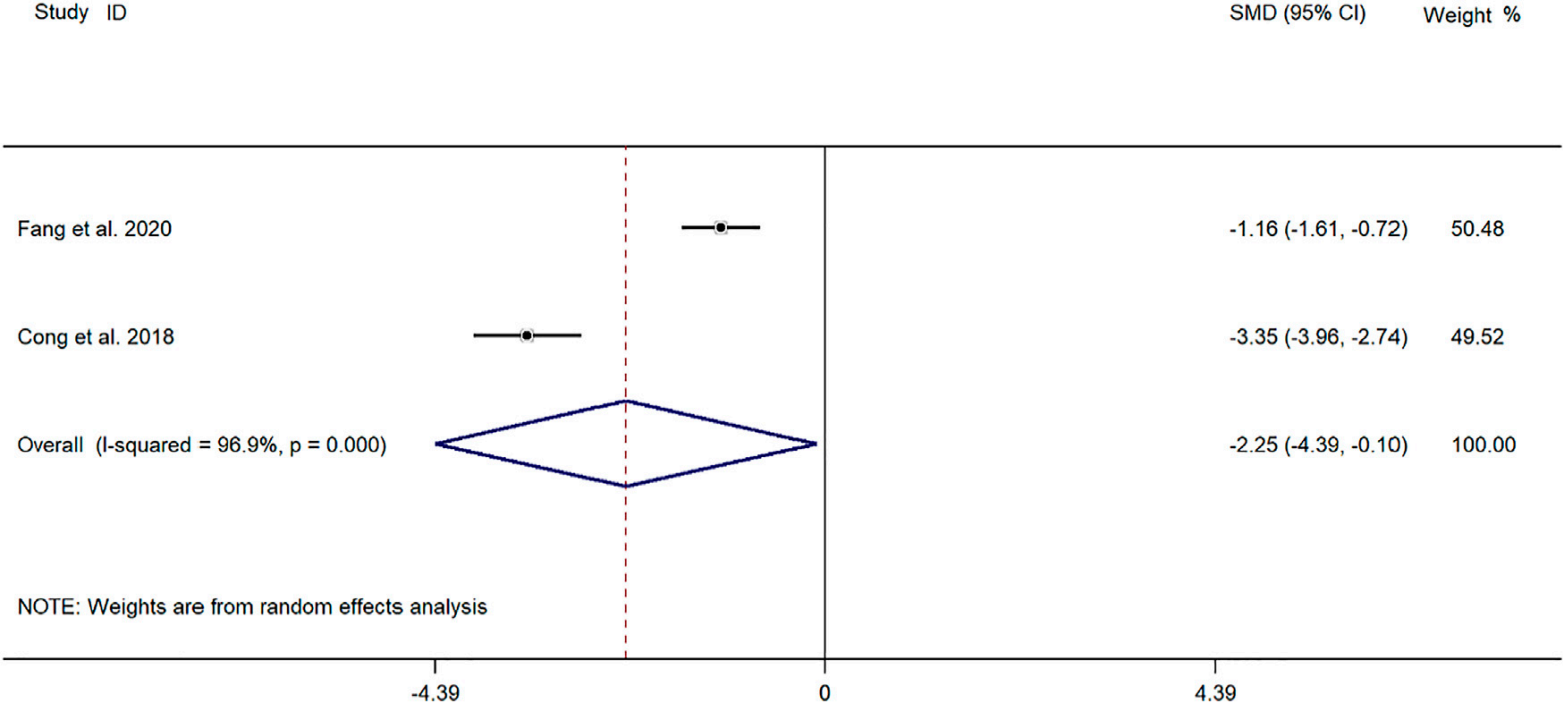
Meta-analysis forest plot comparing the time for relief of cough in the experimental and control group.

Two clinical studies (Cong and Zhu, 2018; Fang and Hang, 2020) reported the cough relief time of SMD in the treatment of *mycoplasma* pneumonia in children. Meta-analysis showed that the index level of cough remission time is obviously heterogeneous (*p* ≤ 0.001, *I* ^*2*^ = 96.9%). Therefore, the random-effects model is selected for Meta-analysis. The results showed that the cough relief time of the experimental group in the treatment of children with *mycoplasma* pneumonia was compared with that of the control group (*SMD* = −2.25, 95%*CI* −4.39 to −0.10, *p* = 0.040), and the difference was statistically significant (Figure 4).

##### Time for Disappearance of Cough

**FIGURE 5:**
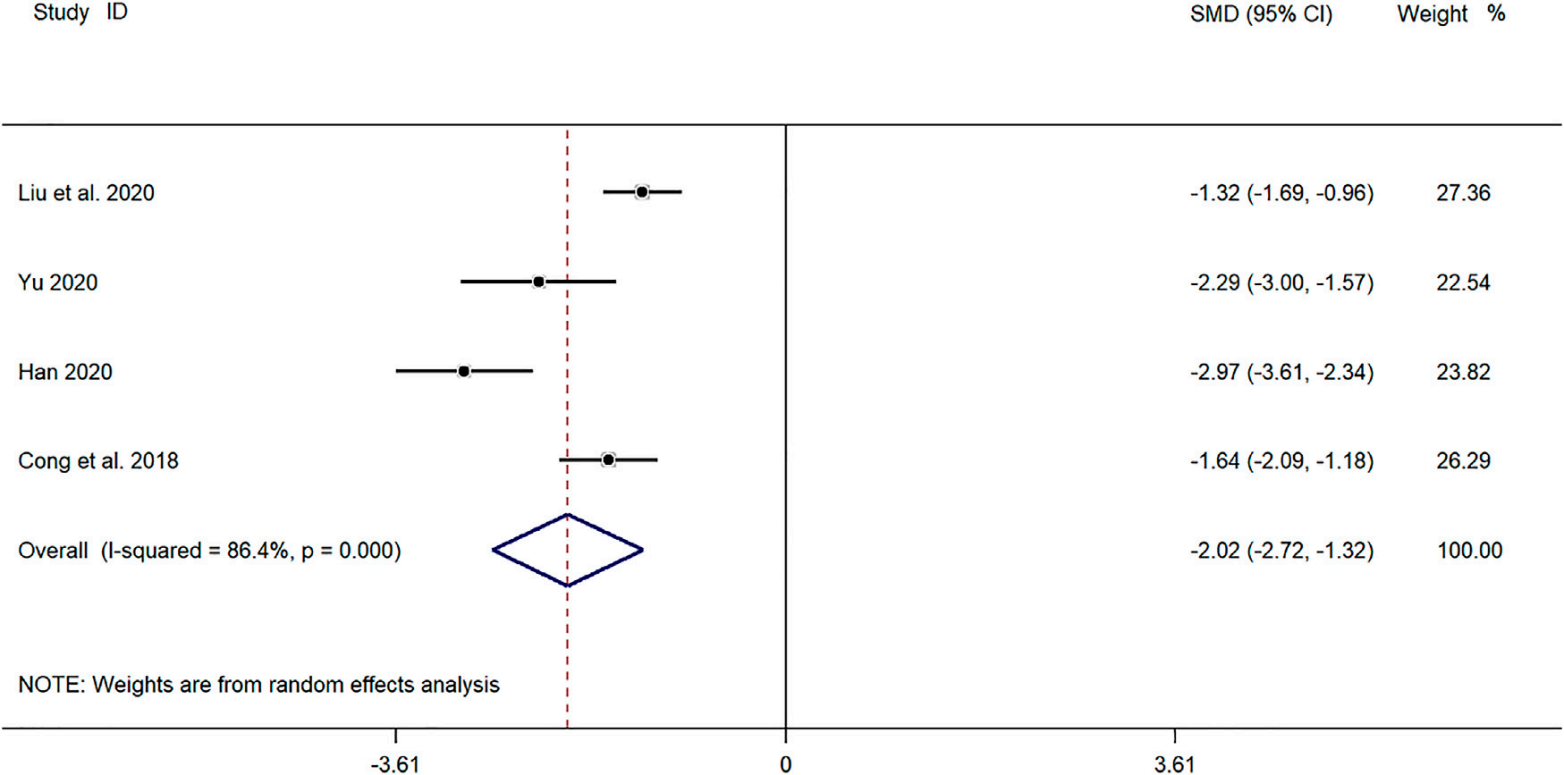
Meta-analysis of forest plots comparing the time for disappearance of cough in the experimental and control group.

Four clinical studies (Cong and Zhu, 2018; Yu, 2019; Han, 2020; Liu et al., 2020) reported the cough disappearance time of SMD in the treatment of children with *mycoplasma* pneumonia. Meta-analysis showed that the index level of cough remission time is obviously heterogeneous (*p* ≤ 0.001, *I* ^*2*^ = 86.4%). Therefore, the random-effects model is selected for Meta-analysis. The results showed that the cough disappearance time of the experimental group in the treatment of children with *mycoplasma* pneumonia was compared with that of the control group (*SMD* = −2.02, 95%*CI* −2.72 to −1.32, *p* ≤ 0.001), and the difference was statistically significant (Figure 5).

##### Time for Disappearance of Lung Rales

**FIGURE 6:**
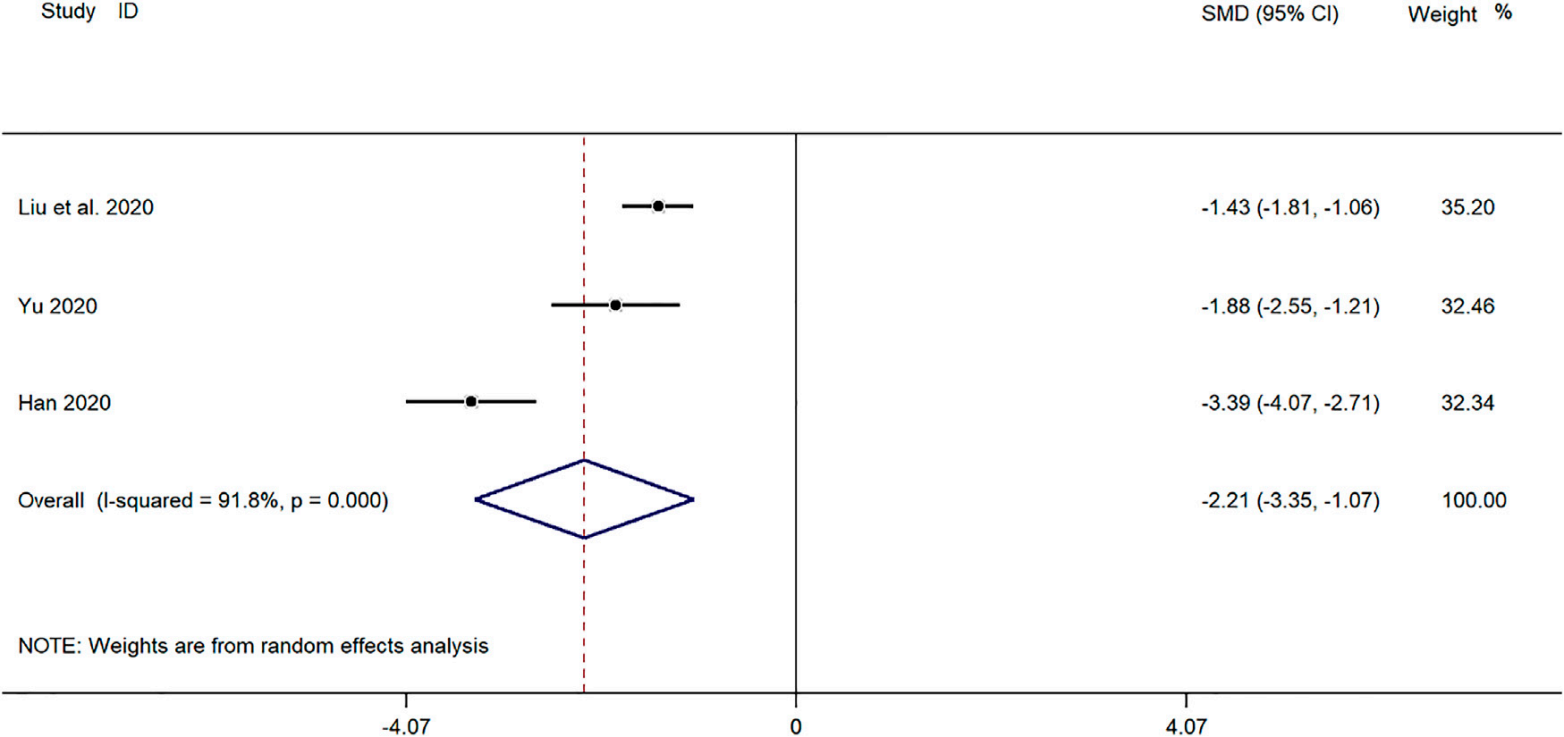
Meta-analysis of forest plots comparing the time for disappearance of lung rales in the experimental and control group.

Three clinical studies (Yu, 2019; Han, 2020; Liu et al., 2020) reported the disappearance time of lung rales in the treatment of *Mycoplasma* pneumonia in children with SMD. Meta-analysis showed that the index level of cough remission time is obviously heterogeneous (*p* ≤ 0.001, *I* ^*2*^ = 91.8%). Therefore, the random-effects model is selected for Meta-analysis. The results showed that the disappearance time of pulmonary rales in the treatment of children with *mycoplasma* pneumonia in the experimental group was compared with that in the control group (*SMD* = −2.21, 95%*CI* −3.35 to −1.07, *p* ≤ 0.001), and the difference was statistically significant (Figure 6).

##### Time for Return to Normal of Chest X-Ray

**FIGURE 7:**
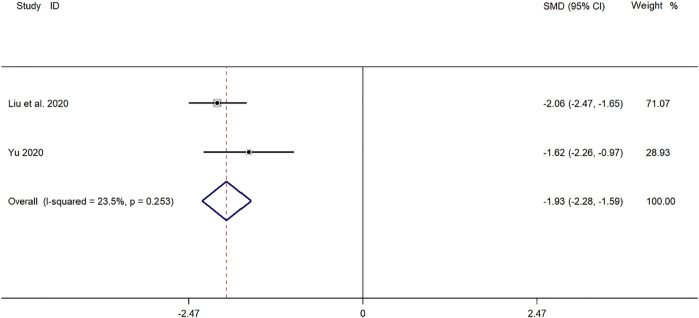
Meta-analysis of forest plots comparing the time for return to normal of chest X-ray in the experimental and control group.

Two clinical studies (Yu, 2019; Liu et al., 2020) reported the time for return to normal of chest X-ray in the treatment of *mycoplasma* pneumonia in children with SMD. Meta-analysis showed that there was no obvious heterogeneity in the index level of chest X-ray recovery time (*p* = 0.253, *I* ^*2*^ = 23.5%). Choose a fixed-effects model for Meta-analysis. The results showed that the disappearance time of pulmonary rales in the treatment of children with *mycoplasma* pneumonia in the experimental group was compared with that in the control group (*SMD* = −1.93, 95%*CI* −2.28 to −1.59, *p* ≤ 0.001), and the difference was statistically significant (Figure 7).

##### Time for Defervescence

**FIGURE 8:**
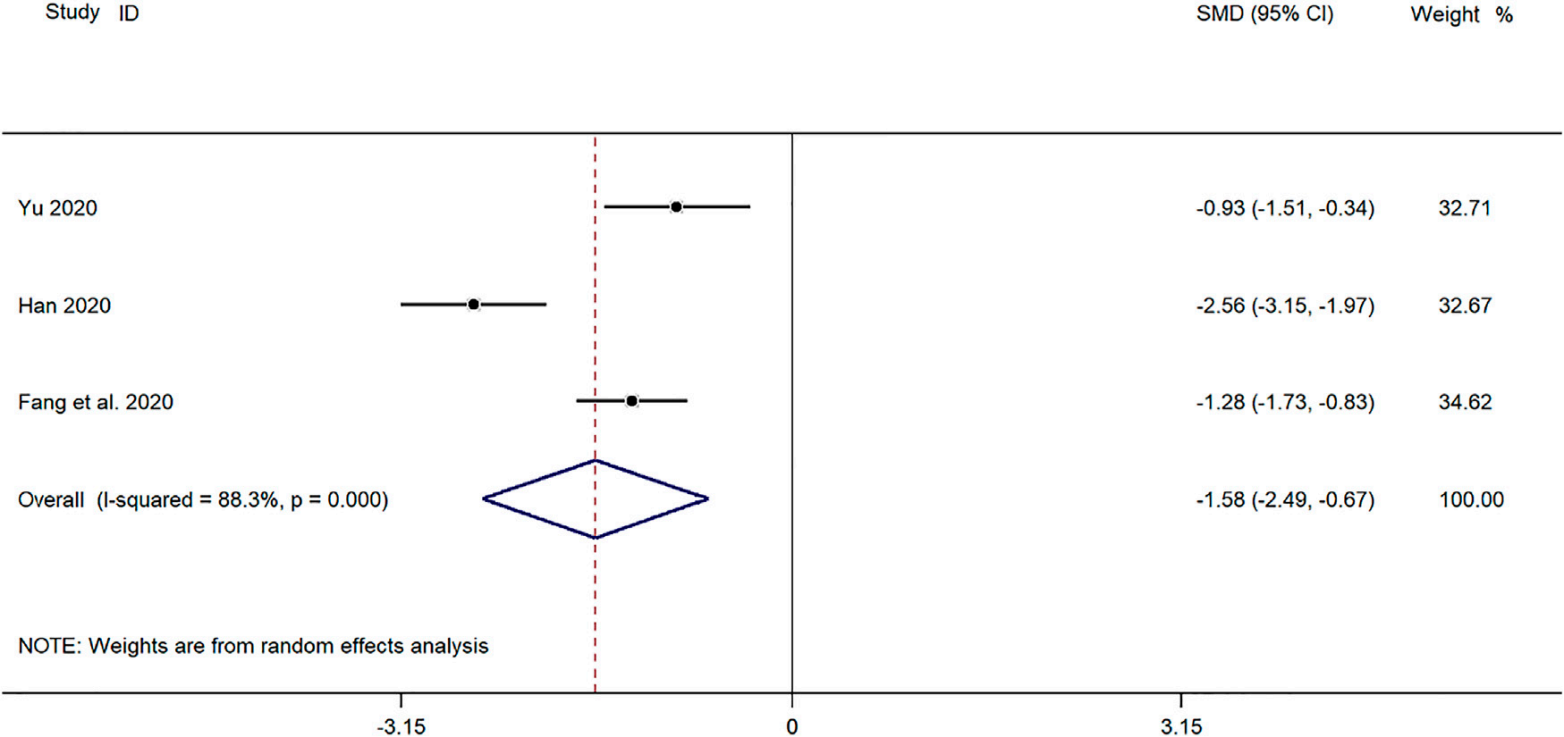
Meta-analysis forest plot comparing the time for defervescence in the experimental and control group.

Three clinical studies (Yu, 2019; Fang and Hang, 2020; Han, 2020) reported the time for defervescence in the treatment of pediatric *mycoplasma* pneumonia with SMD. Meta-analysis showed significant heterogeneity in the level of defervescence time indicators (*p* ≤ 0.001, *I* ^*2*^ = 88.3%). A random-effects model was selected for Meta-analysis. The results showed a statistically significant difference in the time for defervescence in the treatment of pediatric *mycoplasma* pneumonia in the experimental group was compared with that in the control group (*SMD* = −1.58, 95% *CI* −2.49 to −0.27, *p* ≤ 0.001), and the difference was statistically significant (Figure 8).

#### CD3^+^ Cell Levels

**FIGURE 9:**
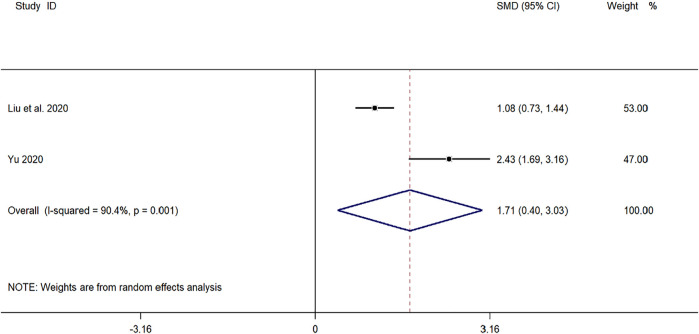
Meta-analysis of forest plots comparing CD4^+^ cell levels in the experimental and control group.

Two clinical studies (Yu, 2019; Liu et al., 2020) reported CD3^+^ levels in the treatment of pediatric *mycoplasma* pneumonia with SMD. Meta-analysis showed significant heterogeneity in CD3^+^ index levels (*p* = 0.001, *I* ^*2*^ = 90.4%). A random-effects model was selected for Meta-analysis. The results showed a statistically significant difference in CD3^+^ cell levels in the treatment of pediatric *mycoplasma* pneumonia in the experimental group was compared with that in the control group (*SMD* = 1.71, 95% *CI* 0.40 to 3.03, *p* ≤ 0.001), and the difference was statistically significant (Figure 9).

#### TNF-α Levels

**FIGURE 10:**
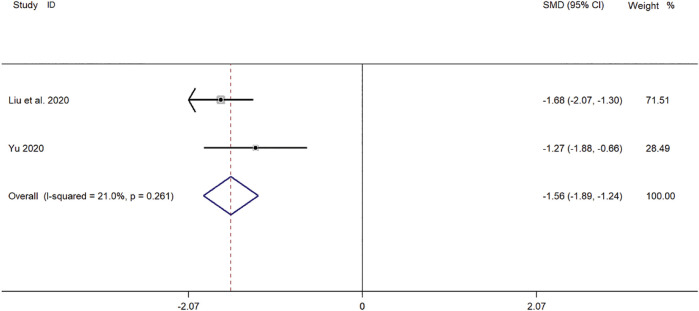
Meta-analysis forest plot comparing TNF-α levels in the experimental and control group.

Two clinical studies (Yu, 2019; Liu et al., 2020) reported TNF-α levels in SMD for pediatric *mycoplasma* pneumonia. Meta-analysis showed significant heterogeneity in TNF-α index levels (*p* = 0.261, *I* ^*2*^ = 21.0%). A fixed-effect model was selected for Meta-analysis. The results showed a statistically significant difference in TNF-α levels in the treatment of pediatric *mycoplasma* pneumonia in the experimental group was compared with that in the control group (*SMD* = −1.58, 95% *CI* −1.89 to −1.24, *p* ≤ 0.001), and the difference was statistically significant (Figure 10).

#### Safety: Incidence of Adverse Reactions

**FIGURE 11:**
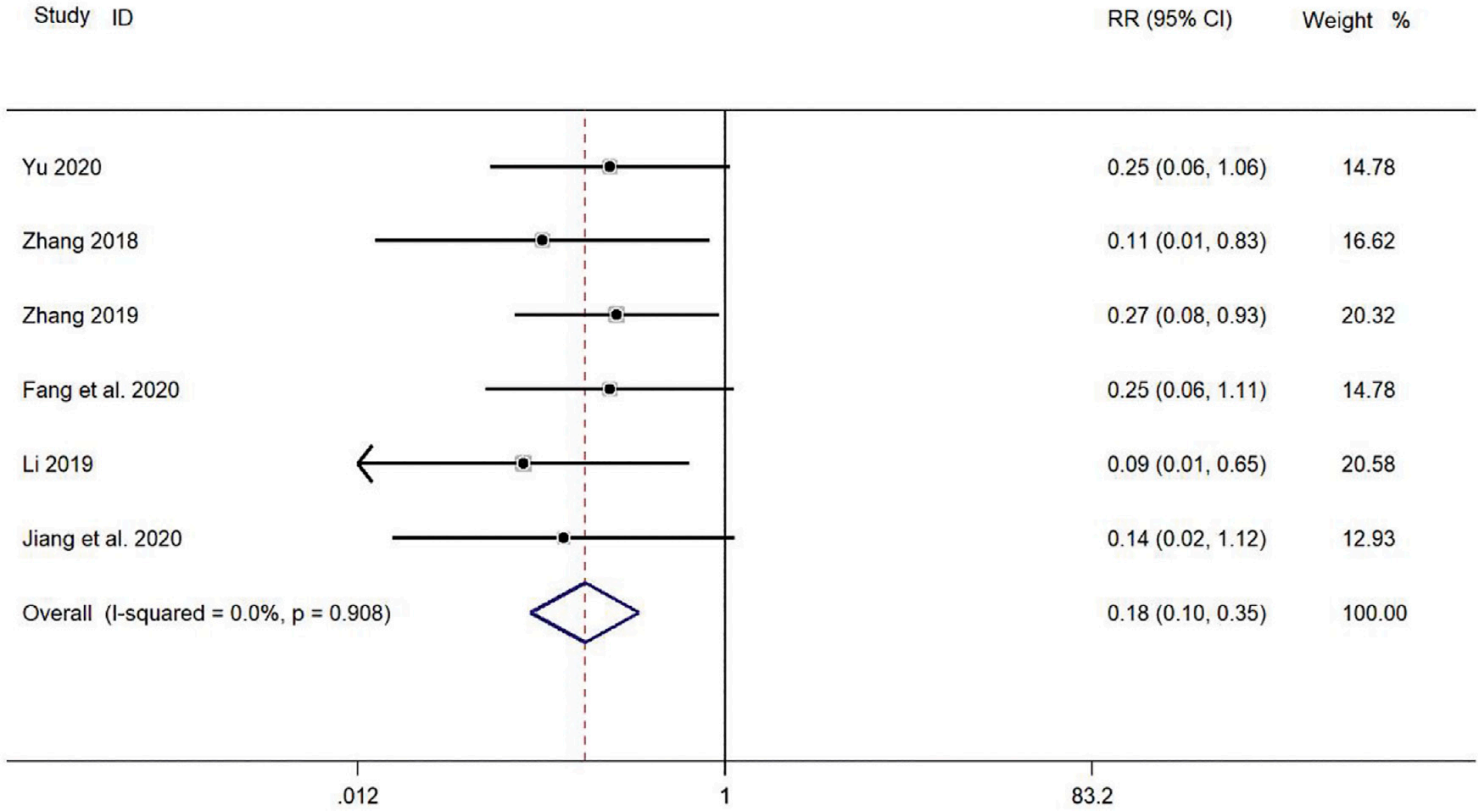
Meta-analysis forest plot comparing the level of incidence of adverse reactions in the experimental and control group.

Six clinical studies (Zhang, 2018; Li, 2019; Yu, 2019; Zhang, 2019; Fang and Hang, 2020; Jiang and Hao, 2020) reported the incidence of adverse reactions in the treatment of pediatric *mycoplasma* pneumonia with SMD. Meta-analysis showed no significant heterogeneity in the level of indicators of adverse reaction incidence (*p* = 0.908, *I* ^*2*^ = 0.0%). Therefore, a fixed-effect model was selected for Meta-analysis. The results showed a statistically significant difference in the incidence of adverse reactions in the treatment of pediatric *mycoplasma* pneumonia in the experimental group was compared with that in the control group (*RR* = 0.18, 95% *CI* 0.10–0.35, *p* ≤ 0.001), and the difference was statistically significant (Figure 11).

### Subgroup Analysis of Relevant Indicators

#### Subgroup Analysis of Different Treatment Duration

**FIGURE 12:**
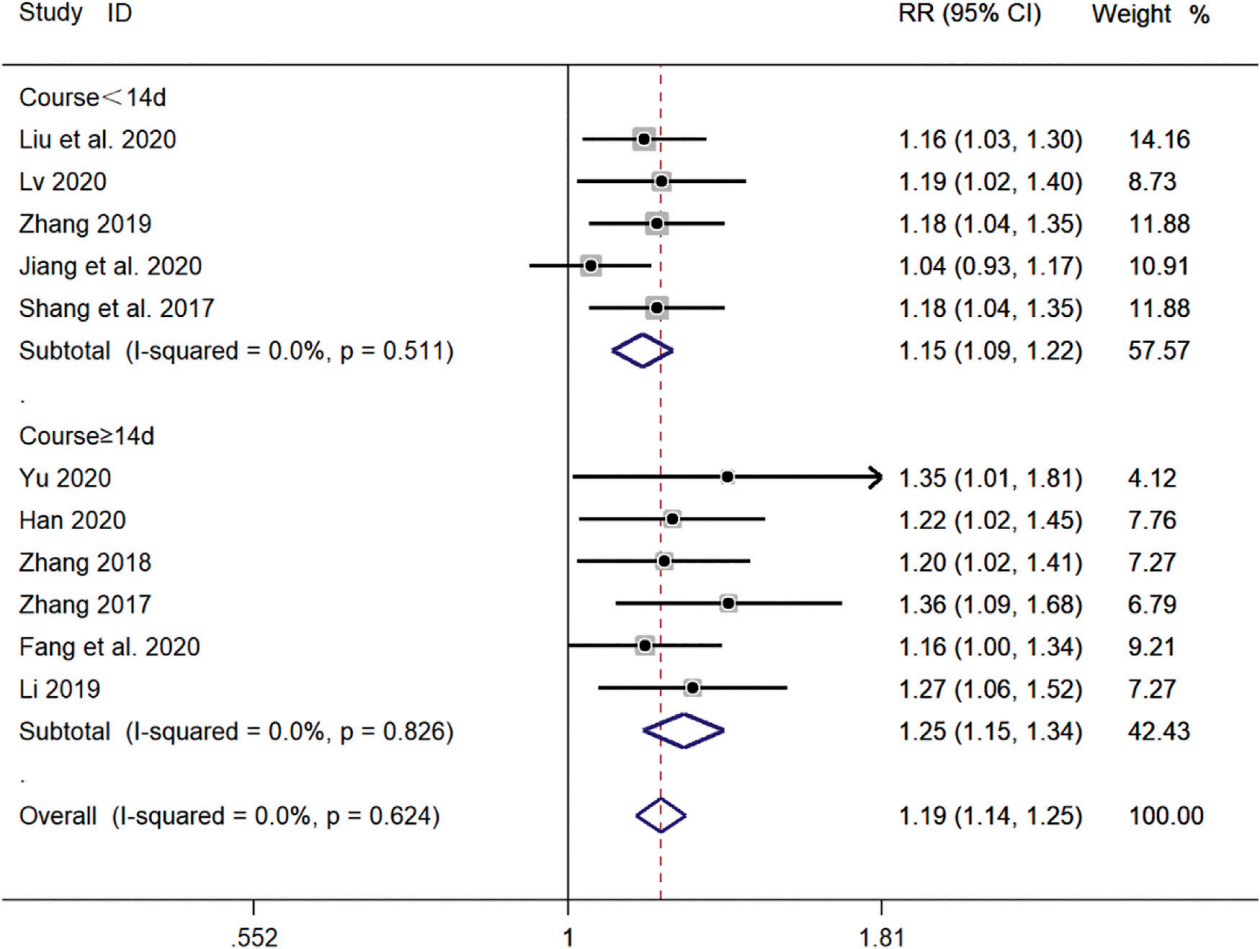
Meta-analysis forest plot comparing the total efficiency level of the experimental group with the control group for subgroup analysis of different treatment duration.

Five clinical studies (Shao et al., 2017; Zhang, 2019; Jiang and Hao, 2020; Liu et al., 2020; Lv, 2020) with a duration of <14 days and six clinical studies (Zhang, 2017; Zhang, 2018; Li, 2019; Yu, 2019; Fang and Hang, 2020; Han, 2020) with a duration of ≥14 days reported the total efficiency level of SMD in the treatment of pediatric *mycoplasma* pneumonia. Meta-analysis showed no significant heterogeneity in the level of the total efficiency index (*p* = 0.624, *I* ^*2*^ = 0.0%). A fixed-effects model was selected for Meta-analysis. The results showed that the total efficiency level of both courses of treatment in the experimental group was significantly higher than that of both courses of treatment in the control group (*RR* = 1.19, 95% *CI* 1.14–1.25, *p* ≤ 0.001), and the difference was statistically significant (Figure 12).

#### Subgroup Analysis of Different Treatment Methods

**FIGURE 13:**
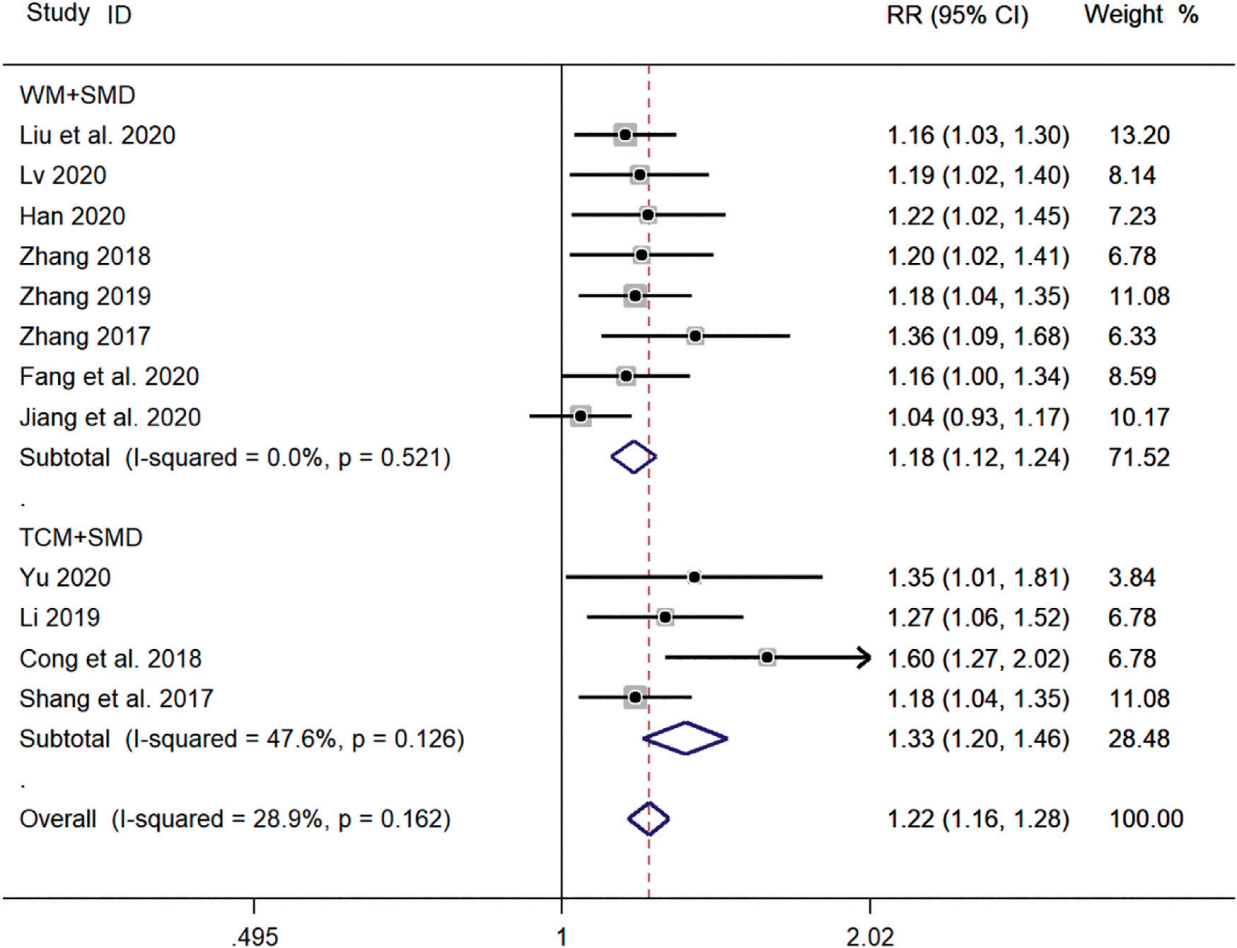
Meta-analysis forest plot comparing the total efficiency levels of the experimental group with the control group for subgroup analysis of different treatment methods. Note: SMD, Shashen Maidong Decoction; WM, Western Medicine; TCM, Traditional Chinese Medicine.

Eight clinical studies (Zhang, 2017; Zhang, 2018; Zhang, 2019; Fang and Hang, 2020; Han, 2020; Jiang and Hao, 2020; Liu et al., 2020; Lv, 2020) using WM + SMD and four clinical studies (Shao et al., 2017; Cong and Zhu, 2018; Li, 2019; Yu, 2019) using TCM + SMD, for a total of 12 publications, reported the total efficiency level of SMD in the treatment of pediatric *mycoplasma* pneumonia. Meta-analysis showed no significant heterogeneity in the level of the total efficiency index (*p* = 0.162, *I* ^*2*^ = 28.9%). A fixed-effects model was selected for Meta-analysis. The results showed that the total efficiency of WM + SMD in the experimental group was significantly higher than that of TCM + SMD in the control group (*RR* = 1.22, 95% *CI* 1.16–1.28, *p* ≤ 0.001), and the difference was statistically significant (Figure 13).

### Sensitivity Analysis

**FIGURE 14:**
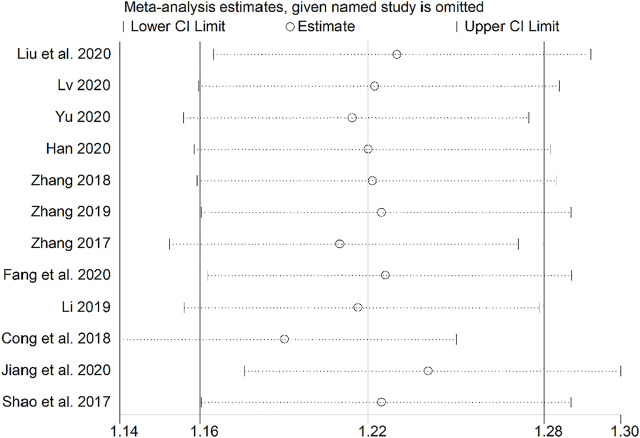
Sensitivity analysis.

Sensitivity analysis was performed using a study-by-study exclusion method, and none of the results changed significantly, suggesting more stable results (Figure 14).

### Publication Bias

**FIGURE 15:**
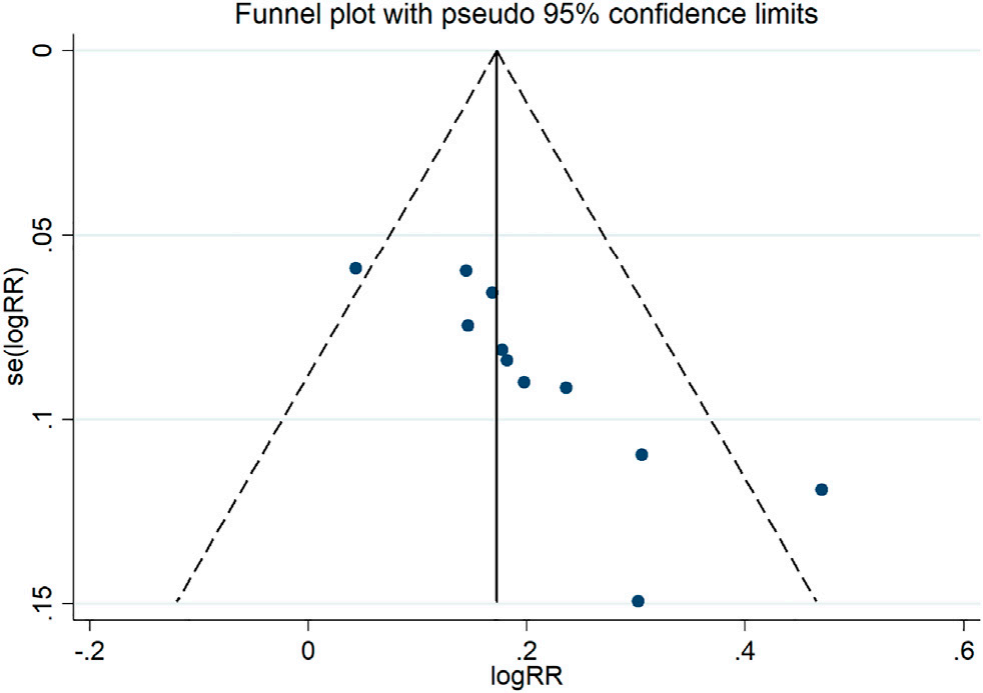
funnel plot.

**FIGURE 16:**
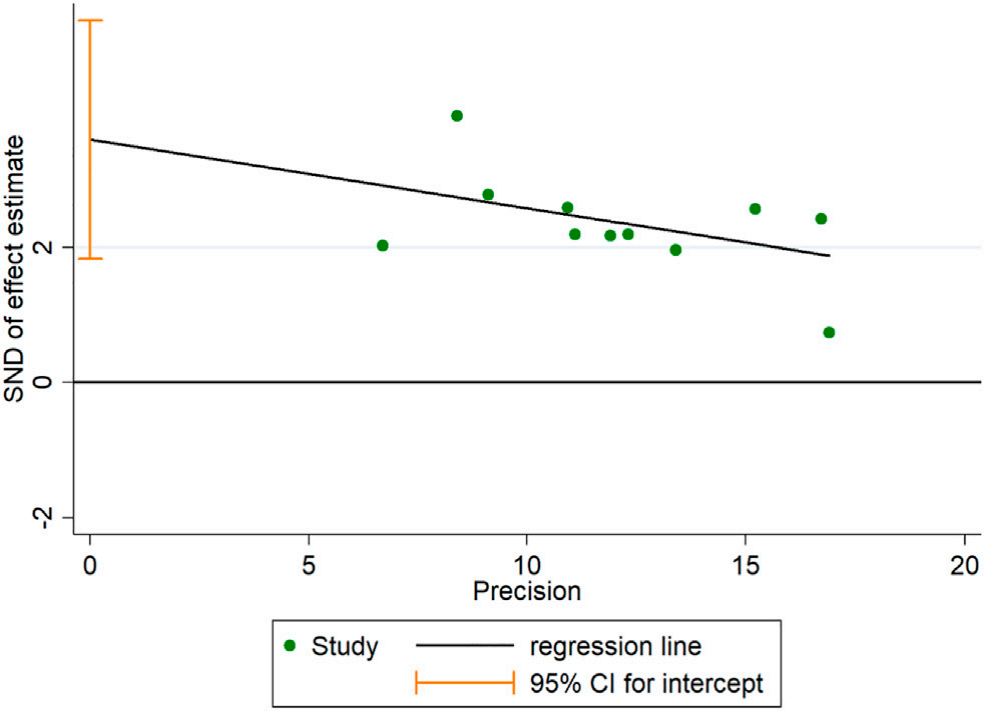
Egger’s test.

A funnel plot for publication bias test for the outcome indicator of the total efficiency showed an asymmetric left-right distribution across study sites (Figure 15), with an Egger’s test result of *p* = 0.001 (Figure 16), suggesting the existence of publication bias.

## Discussion

*Mycoplasma pneumoniae* (MP) infections occur frequently in different countries and regions, and bronchitis and pneumonia are the most common clinical diagnoses associated with MP infections (Defilippi et al., 2008). MP is the main pathogen of MPP, and modern medical studies have confirmed that macrolide antibiotics can significantly improve the cure rate of this disease (Qiu et al., 2020). However, over the past decade, macrolide resistant *Mycoplasma pneumoniae* has shown epidemic trends worldwide, the most severe of which is in Asia, where prevalence rates range from 13.3 to 100%, and can even reach 90% in Japan at certain times of the year (Chen et al., 2020), and nearly 80% in Korea (Lee et al., 2021). In China, more than 85% of *Mycoplasma pneumoniae* (MP) strains in pediatric patients have been reported as macrolide-resistant *Mycoplasma pneumoniae* (Guo et al., 2019). This bacterium is also one of the most common causes of CAP infection (Atkinson et al., 2008). The clinical features of patients are mainly headache, cough, and sore throat, and the short onset and worse condition are the characteristics of their disease, which may manifest as dyspnea, wheezing, and coughing when it worsens, and if left untreated, can lead to substantial organ lesions that can recur (Han et al., 2018), so the prevention and treatment of this disease is necessary and urgent.

SMD is mainly composed of *Glycyrrhiza uralensis* Fisch. ex DC (Fabaceae; *Glycyrrhiza uralensis* radix & rhizome), Polygonatum odoratum (Mill.) Druce (Asparagaceae; *Polygonatum odoratum* rhizome), *Adenophora stricta* Miq. (Zingiberaceae; *Alpinia oxyphylla* fruit), *Vicia lens* subsp. Lens (Fabaceae; *Vicia lens* subsp. Lens seed), Ophiopogon japonicus (Thunb.) Ker Gawl. (Asparagaceae; *Ophiopogon japonicus* radix), *Trichosanthes kirilowii* Maxim. (Cucurbitaceae; *Trichosanthes kirilowii* radix) and *Morus alba* L. (Moraceae; *Morus alba* leaf). We have finally determined the preparation method (Yan et al., 2021) and administration method (He et al., 2021) of SMD by consulting the literature. Preparation method: Weigh 11.19 g each of Sha Shen and Mai Dong, 5.60 g each of Sang Ye, Tian Hua Fen, and Sheng Bian Dou, 7.46 g of Yu Zhu, 3.73 g of Gan Cao, add 1 L of water, decoct until the remaining 400 ml, filter the medicinal residues, and get the SMD. Method of administration: Take 200 ml of decoction, take it warmly, 2 times a day, once in the morning and once in the evening, 1 dose a day. The specific stopping or reducing time depends on the condition of the disease (He et al., 2021). It is mainly used in traditional Chinese medicine to treat “yin deficiency, dryness, heat and lung injury, fever and cough” (Yang and Zhou, 2019). By studying the HPLC fingerprint of SMD, the significance of the quality evaluation of SMD has been improved (Yan et al., 2021). Modern research shows that the pharmacological effects of SMD are mainly reflected in anti-inflammatory, immune enhancement, gastric mucosal protection, inhibition of gastric hyperactivity, anti-oxidation, anti-tumor, etc., (Gao et al., 2020), and its main components such as *Adenophora* polysaccharides (Zheng et al., 2012), *Ophiopogon japonicus* polysaccharide (Sun et al., 2021), Mulberry leaf polysaccharide (Wang and Xiao, 2020) and Trichosanthin (Ouyang and Wu, 2021) can regulate the inflammatory environment. Therefore, it can provide reliable preclinical evidence for the treatment of *mycoplasma* pneumonia in children with SMD.

Systematic review and meta-analysis is a widely accepted research method and is at the top of the hierarchy of clinical evidence (Izzo et al., 2016). However, there is no clinical evidence evaluating the safety and efficacy of SMD in the treatment of pediatric *mycoplasma* pneumonia in current evidence-based medical studies.

According to the analysis of the overall research results of mate analysis, there is an interesting phenomenon: when the experimental group contains SMD, the relevant index levels are better than those of the control group; when both the experimental group and the control group have SMD, The observation group treated with traditional Chinese medicine has better-related index levels than the control group treated with western medicine. This can at least explain that SMD and traditional Chinese medicine are more effective than western medicine in the treatment of childhood *mycoplasma* pneumonia.

In this study, a total of 12 clinical studies were included, and a total of 1,127 patients with *mycoplasma* pneumonia were treated with SMD, and the levels of total effective rate, time to disappearance of cough, time to relief of cough, time to fever reduction, time to chest film normalization, T lymphocyte subpopulation (CD3^+^) and tumor necrosis factor-α (TNF-α) were analyzed, and the results showed that the clinical efficacy of the observation group treated with SMD was significantly The results showed that the clinical efficacy of the observation group treated with SMD was significantly better than that of the control group, and the levels of all indexes analyzed were statistically significant. The results of the subgroup analysis of the different treatment courses showed that the treatment duration could be extended to maximize the effect when treating *mycoplasma* pneumonia, and the subgroup analysis of the different treatment methods showed that the use of TCM and SMD in combination was more effective than the use of Western medicine and SMD in combination, which highlights the important role of TCM in the prevention and treatment of the disease.

Among all 12 studies, six studies reported adverse reactions. Meta-analysis was performed on the observation group and the control group. The results showed that the use of SMD in the treatment of *mycoplasma* pneumonia can significantly reduce the incidence of adverse reactions after treatment. Modern pharmacological studies have found that Mai Dong (*Ophiopogon japonicus* (Thund.) Ker Gawl.) (Fan et al., 2020), Yu Zhu (*Polygonatum odoratum* (Mill.) Druce) (Meng et al., 2020), Tian Hua Fen (*Trichosanthes kirilowii* Maxim.) (Cao, 2017), Gan Cao (*Glycyrrhiza uralensis* Fisch. ex.DC) (Deng et al., 2021), etc., have immunomodulatory functions, which can improve disease resistance and treatment tolerance of children, and reduce the treatment Toxic and side effects reduce the incidence of adverse reactions.

## Limitations

This study has the following limitations: first, the number of studies included in this study is small, and there is an uncertainty in the assessment of clinical efficacy of SMD in the treatment of pediatric *mycoplasma* pneumonia. Second, the search text type of this study was limited to Chinese and English databases, and did not include Japanese and Korean databases, which may result in the exclusion of some high-quality articles. In addition, for the risk of bias assessment of the included studies, only six papers specifically described the randomization method, and the risk of bias for the others was not known, which also led to some risk of bias in this study. Finally, the quality of the included clinical studies was not high, which also suggests that the next more in-depth studies need to include higher-quality literature to provide more reliable clinical evidence to support the rational clinical application of SMD.

## Conclusion

The results of the systematic evaluation showed that SMD significantly reduced the level of outcome indicators such as time to symptom relief. The analysis of different subgroups can further illustrate the characteristics of SMD in the treatment of pediatric *mycoplasma* pneumonia. SMD can improve the clinical symptoms of pediatric *mycoplasma* pneumonia. The efficiency of SMD in the treatment of *mycoplasma* pneumonia in pediatric patients was high, and the incidence of adverse effects was low. In general, random-effects models yield conservative conclusions, and the final combined results of random-effects and fixed-effects models do not differ significantly when heterogeneity is small, and are more effective when used with large heterogeneity. Therefore, random-effects model analysis can be used to reduce the variation for clinical indicators with fewer included studies. In general, the results of the meta-analysis are sufficient to show that it is effective at least in the treatment of *mycoplasma* pneumonia in children, whether combined with WM or alone. The quality assessment of the included literature is risky to a certain extent, because some factors of the assessment are uncertain. SMD is a traditional Chinese prescription and is widely used and researched in China. Therefore, most of the included literature is in Chinese and the original research data are from the clinic. This can provide reliable evidence for the conclusion of this study. The effectiveness of SMD in treating *mycoplasma* pneumonia in children is credible, however, the main components and specific mechanism of the efficacy of SMD are unknown, which will be a major research in the future, not only SMD, but also the mechanism of action of more herbal compounds need to be explored, which is also the core direction of the development of Motherland Medicine. For future clinical studies on *mycoplasma* pneumonia, experimental protocols should be designed more scientifically and rationally to reduce the risk of bias and improve the quality of evidence, to further evaluate the efficacy of SMD in the treatment of pediatric *mycoplasma* pneumonia and its scientific and feasibility in clinical studies.

## Data Availability

The raw data supporting the conclusions of this article will be made available by the authors, without undue reservation.

## Abbreviations

ACU, acupuncture, AZM, azithromycin, CAP, community-acquired pneumonia, C, control group; CT, conventional therapy, T, experimental group; F, female; M, male; MA, macrolide antibiotics; MP, mycoplasma pneumoniae; MPP, mycoplasma pneumonia; MA, macrolide antibiotics; TCM, traditional chinese medicine; WM, western medicine; SMD, Shashen Maidong decoction; SGD, Shaoyao Gancao decoction; YYQFD, Yangyin Qingfei decoction.
